# Automated Sample Storage in Biobanking to Enhance Translational Research: The Bumpy Road to Implementation

**DOI:** 10.3389/fmed.2019.00309

**Published:** 2020-01-09

**Authors:** Loes Linsen, Kristel Van Landuyt, Nadine Ectors

**Affiliations:** AC Biobanking, University Hospitals Leuven, Leuven, Belgium

**Keywords:** biobank, automation, sample storage, quality, temperature mapping, qualification, translational research

## Abstract

The low reproducibility of biomarker research is a major holdback for the translation of research results to the bedside. Sample integrity has been identified as a key factor that contributes to improved reproducibility. The key mission of biobanks is to ensure that all activities and materials are managed according to standardized procedures and best practices to ensure and preserve sample integrity. When handling large numbers of biospecimens automation of sample handling and storage is often the method of choice to maintain and improve sample integrity. In December 2013, the centralized Biobank of the University Hospitals and the Catholic University of Leuven (UZ KU Leuven) decided to implement automated systems for sample storage and retrieval, one for storage at −20°C and one for storage at −80°C. Here we describe the extensive process of installation, acceptance, validation, and implementation of these two systems. Overall it took about 4 years to effectively take the systems into production. Multiple issues resulted in the delayed implementation, with labware change, quality of the initial installation, and misunderstanding of biobank concerns being the most impacting. Significant effort in terms of time and resources from both the automated store supplier as well as the biobank itself was needed to achieve a successful implementation. Within 15 months of actual integration in the biobank workflow, over 63 k samples were placed into the systems. Actual hands-on sample handling and retrieval times were substantially reduced, although this implied the shift of dedicated personnel time from the researchers' laboratories to the biobank. With the successful implementation of automated frozen sample storage systems, the centralized UZ KU Leuven Biobank is now also able to efficiently support large-scale translational research.

## Introduction

The low reproducibility of biomarker research is a major holdback for the translation of research results to the bedside (1, 2). The integrity and quality of the biospecimens used for research, has been identified as one of the key factors that contribute to improved reproducibility (3). Biobanks play a vital role there, because they are the custodians of the biospecimens required for research (4). As such, it has been recognized that the biobanks are a cornerstone of precision medicine (5). The key mission of biobanks is to ensure that all activities and samples are managed according to standardized procedures and evidence-based best practices to ensure and preserve biospecimen integrity (6). Several initiatives of biobank harmonization and standardization have ultimately cumulated into the publication of the biobank standard ISO 20387:2018, intended to ensure quality, fitness-for-purpose, and reproducibility in biobanking to facilitate translational research progress (7).

It is a well-known fact that up to 70% of the analytical errors are due to variations in the pre-analytical phase in the clinical laboratory environment (8). In the context of biospecimens and biobanking, the pre-analytical phase covers all processes between the collection of the sample until it is removed from storage for analysis (9). When handling large numbers of biospecimens for processing or analysis, automation of tasks is often the logical next step to reduce labor costs and increase sample throughput. There is a consensus in bioanalytical laboratories that automation also shortens method development time and improves biosafety and quality of data and samples (10). Automation of non-analytic functions has even greater importance, since it is recognized that non-analytic errors are more significant than analytic errors in terms of general laboratory quality (11). Tasks that are repetitive and monotonous, such as sample storage and retrieval, are the most prone to human errors but are also more easily automatable (11). Furthermore, biospecimen retrieval from frozen storage often is performed manually, thereby frequently exposing samples to large temperature fluctuations which can introduce variation in the sample and may have a detrimental effect on its quality (12). Automating the biospecimen storage and retrieval process reduces the exposure of samples to temperature variation and is therefore an efficient option to preserve the sample quality for sustainable translational research.

The Biobank of the University Hospitals and the Catholic University of Leuven (UZ KU Leuven) was founded in 2008 in response to changing national regulation. Its current main objective is to centralize the storage of human bodily material for scientific purposes and make it available for intra- and extramural research projects. The biospecimens are collected and processed by different partners of the biobank, such as the laboratories for pathology, clinical chemistry, and molecular diagnostics, but also by various research groups belonging to the University and University Hospital. As a result, a plethora of sample types and associated containers have to be accommodated at different conservation conditions. In an effort to optimize the storage of frozen liquid biospecimens such as serum, urine and plasma, it was decided in December 2013 to implement two automated systems for sample storage and retrieval, one for storage at −20°C and one for storage at −80°C.

Regulatory standards such as those from the biobanking ISO 20387:2018 require a formal qualification and validation of an automated frozen storage system before taking it into production, as well as frequent reassessment of its performance of both the automation and temperature component (13). However, the validation requirement is subjective and leaves the implementing biobank without specific criteria to tackle this process. Additionally, our extensive literature searches revealed no information regarding guidelines for automated storage system validation and qualification. Furthermore, no procedures for customer validation were available from the manufacturer. We therefore developed a validation/qualification method for automated frozen sample storage systems, based on the implementation of two systems (one at −20°C and one at −80°C) at the UZ KU Leuven Biobank. Furthermore, we describe the challenges encountered and the solutions created in the process of implementation, allowing others to learn from our experience.

## Materials and Methods

### Automated Sample Store Structure

The Sample Store I (−20°C) and the Biostore (−80°C) (Brooks Life Sciences, Manchester, UK) have a similar main design which is described in the Supplementary Material.

### Automation Operational and Performance Testing

The system qualification and assessment test (SQAT) was designed to assess the operational functionality of the system used in daily routine and used in the operation qualification of the system: input, scanning, placement in storage, reformat of samples from standard density to high density trays, picking of samples from high density to standard density trays, output. The SQAT routine was devised by the UZ Leuven super-user, based on previous experience of IQ/OQ/PQ validations of analytical equipment and standard practice thereof, as no information was available in literature or from the manufacturer for validation of automated storage systems. The SQAT was performed as follows: one standard tube rack with 96 tubes filled with 900 μl saline was put on a standard density tray together with five empty tube racks and put in the input/output module for input. Correct identification of tray, rack and sample barcodes by scanning was verified using the user software interface. Upon successful input, an order for sample reformat was created. Tray loading and sample reformat in and tray unloading from the cherry picker, as well as pulling trays from and putting trays back in storage were observed. Storage locations were registered. Pick retry and place retry rates were recorded before and after reformat for continuous monitoring and should remain below 10% for the Biostore and below 5% for the Sample Store I. Upon successful reformat, an order to pick 50–100% of samples from the input was generated, where the same parameters as described for the reformat order were registered. Upon completion of the pick order, an order was generated to output and scan the tray holding the picked samples. Barcodes of outputted trays, racks and samples were verified. The SQAT takes 15 and 20 min to complete for the Sample Store I and the Biostore, respectively. To measure reformat and picking times, the relevant parts of the SQAT were used and adapted to contain the number of samples required for the tests (1–10–100–1,000 tubes). Time was calculated from start time of the order to end time of the order as defined in the user interface.

Formal performance qualification was performed through empty runs mimicking the first customers' sample flow. Based on current practices at the time of the test, it was estimated that 125 frozen samples would be inputted in one batch daily and about 25 samples would be outputted in one batch weekly. During a 2-week period these movements were simulated with saline-filled tubes for both the Sample Store I and the Biostore and recorded using the SQAT document. Formal qualification criteria were: no user-intervention required to complete the test apart from the default use. Identification of tubes entering and exiting the automated stores concordant with independent identification (FluidX Perception scanner), pick and place retry ratio's remained below 5 and 10% for the Sample Store I and Biostore, respectively.

### Temperature Homogeneity Testing

Temperature mapping was performed using a protocol modified from the Energy Star Program Requirements for Laboratory Grade Ultra-low Temperature Freezers (14). Fifteen PT 100 air temperature monitoring devices (TMD) (Testo 184 T4 data logger, Testo, Lenzkirch, Germany) were placed on high density trays and inputted into the automated stores according to positions for environments with a storage volume between 2 and 20 m3 as stipulated in the NF X 15-140 standard. The TMDs are distributed in three planes, one located at the highest possible tray location accessible for samples, one at the geometric center and one at the lowest possible tray location accessible for samples. For the upper and lower planes, the TMDs are positioned in each corner and at the center. For the geometric center plane, TMDs are positioned at the four midpoints between all corners and at the center. TMDs were left to acclimatize for at least 5 h before starting the test. Temperature was measured each minute for 24 h. During the 24 h test period, door opening and closing impact was performed by opening the inner doors between the input/output module and the store for 45 s (followed by opening of the tile wall for 10 s in the Biostore) and repeating this schedule 3 times once per hour for a period of six consecutive hours. The average store temperature was calculated from all TMDs for the 24 h measurement period. The peak variance was calculated as the difference between the maximum and minimum temperatures measured across all TMDs over the 24 h measurement period. The stability was determined as the difference between the maximum and the minimum temperature measured by an individual TMD over the 24 h test period. The uniformity was calculated as the difference between the maximum and minimum temperature measured inside of the unit at any given time. The impact of door opening and closing was assessed by calculating stability and uniformity for a 3 h period starting with the first door opening exercise and for a 3 h period, starting 3 h after the last closing of the door. Warm spot and cold spot are defined by the TMDs showing on average the highest or the lowest temperature over the 24 h measurement period, respectively. The data were exported to comma separated files using the Testo Comfort Software (Testo, Lenzkirch, Germany). All calculations were done using Microsoft Excel 2016 (Redmond, Washington, U.S.).

## Results

### On-Site Installation of Automated Sample Stores and Site-Acceptance Testing

In 2013, the Sample Store I (−20°C sample storage) and Biostore I (−80°C sample storage) from Brooks were selected for installation at a dedicated site of the UZ KU Leuven Biobank. About 1.5 years after purchase, the two systems were ready for a site-acceptance test (SAT). The 13-point SAT was drafted by the manufacturer and was a high level assessment of the systems' functionality. The Biobanks' expectations/criterion to pass the SAT was that all elements would be cleared consecutively on the first attempt. While the Sample Store SAT passed without problems, the Biostore SAT was only passed by repeatedly attempting the individual elements and making modifications to the system in between at that time. As a result, the Biostore SAT was not signed off by the UZ KU Leuven Biobank.

Following the initial SAT, the Biostore system presented persistent problems regarding automation and cooling performance afterwards. In an effort to address these issues, the Biobank decided to appoint an internal dedicated super-user whose main focus was to obtain a deeper understanding of the system and design a structured approach for improvement. To chart the robotics issues, a system qualification and assessment test (SQAT) was devised by the Biobank, assessing the four main components of routine use: sample input and scanning, sample reformat from standard density to high density trays, sample picking from high density to standard density trays and sample output and scanning. Table 1 shows that over a period of 7 months, 30.2% of these tests failed (*n* = 196), with a 13.5% failure rate observed for overall activities, identifying the SQAT as a simple but effective tool to assess the systems' performance. The main causes for failure were due to imaging issues, tube picking problems and mechanical crashes. Failures occurred irregularly and the type of failure was unpredictable.

**Table 1:** Overview of causes for automation failure of Biostore.

		**Tests** **(*n* = 43)**		**Total activity** **(*n* = 414)**	
**Element**	**Cause**	***n***	**%**	***n***	**%**
Robot	Minicrash	3	7.0	4	1.0
Imaging	Tube not detected	2	4.7	21	5.1
Imaging	Failed image integrity	1	2.3	5	1.2
Picker	Picker calibration	4	9.3	11	2.7
Picker	Tube on tube error	2	4.7	3	0.7
IT	Software crash	0	0.0	1	0.2
Other		1	2.3	11	2.7
**Totals**		13	30.2	56	13.5

In addition to the automation issues, the Biostore cooling system also did not show the expected robustness, evidenced by one of the redundant cooling units failing at weekly switch-over due to recurrent high-pressure problems (32 times across 6 months). Furthermore, minor incompletions of the installation resulted in an increased frost build up, leading to imaging problems explaining some of the issues summarized in Table 1.

Overall, these observations suggested a suboptimal quality of the original installation. Corrective actions were taken by the manufacturer upon increasing pressure by the Biobank, requiring significant efforts from both the manufacturer and the biobank to rectify the situation over a period of 2 months. After completion of the works in December 2016, a second SAT was performed which was passed on first attempt, without any additional modifications needed, declaring the automated stores ready for validation by the Biobank. This was confirmed by a post-SAT2 SQAT failure rate of <1% (data not shown).

### Sample Storage Tube Integrity Testing

At the same time as the implementation of the corrective measures, the labware showed clear fractures in the wall of the tubes when these were filled with the allowed amount of water and frozen in either the Biostore or the Sample Store I (brand 1, 1 ml high density tubes, 2D barcoded bottom, internal threaded caps). Experiments were conducted to assess the extent and cause of the problem. Similar tubes obtained from two additional manufacturers were subjected to the same conditions (brand 2, brand 3). Maximum fill volumes as defined by the manufacturers was ≤920 μl. The tubes were filled with 900–970–1,000 μl of distilled water, saline, serum or a suspension of cells in 10% serum and subsequently frozen in either the Sample Store or the Biostore. Tubes were deliberately overfilled (970–1,000 μl) to simulate pipetting mistakes made by customers providing their pre-filled sample tubes to the biobank.

Fractured tubes were only observed when tubes were filled with distilled water. As shown in Table 2, all brands tested showed this behavior when the tubes were frozen in the high-density tray at −80°C, even when the fill volume was within the manufacturers' range, except for brand 1. However, this brand also showed fractures when frozen in the Sample Store at −20°C while the other brands did not show any fractures in that condition. Clearly, the different tubes behaved differently to different conditions, which might be explained by the composition of the tube plastic/polypropylene. As the cherry picker module of the installed stores was specifically configured to the tube type selected by the UZ KU Leuven Biobank (brand 1), these findings were presented to the manufacturer. Based on the manufacturers' test results, a different tube type was suggested (brand 1, 1 ml high density tubes, 2D barcoded bottom, external threaded caps), with the same tube diameter albeit 6.6 mm shorter, but still compatible with the existing cherry picker module given some minor modifications to the pick head height. These tubes were subjected to the same tests as described above and indeed showed no formation of fractures in any of the tested conditions. When overfilled, the caps were ejected from some of the tubes due to increased internal tube pressure upon ice formation (75% for 970 μl; 100% for 1,000 μl). This caused problems with the reformat/picking functionality of the cherry picking module. To prevent this problem, a volume assessment routine at sample intake was implemented. Furthermore, the routine input approach for non-frozen samples into the automated stores was set to freeze the samples overnight in the standard density racks before reformatting into the HD tray. This also allowed a visual check of cap presence to occur before any reformat/picking action was started. Although this resulted in a reduction of the volume stored, it was decided to accept the new tube type. The necessary modifications to the cherry picker module were executed in parallel with the adjustments required to improve the overall installation. The performance of the modification was qualified during the second SAT.

**Table 2:** Proportion of fractured tubes upon freezing in Biostore and Sample Store I at different fill volumes of distilled water.

	**Fill volume (μl)**	**Brand 1 (HD)**	**Brand 2 (HD)**	**Brand 3 (HD)**	**Brand 1 (SD)**	**Brand 1 (HD—external)**
Biostore	900	0.0%	66.7%	83.3%	8.3%	0.0%
	970	70.8%	33.3%	50.0%	45.8%	0.0%
	1,000	100.0%	66.7%	83.3%	95.8%	0.0%
Sample Store	900	37.5%	0.0%	0.0%	0.0%	0.0%
	970	16.7%	0.0%	0.0%	0.0%	0.0%
	1,000	41.7%	0.0%	0.0%	100.0%	0.0%

*HD, samples frozen seated in high density tray; SD, samples frozen seated in standard density SBS racks*.

### System Qualification and Validation

Upon formal acceptance of the installation, an additional set of assessments was performed to qualify the automated stores for the intended use in the UZ KU Leuven Biobank. The automation aspect was tested on several levels with the main criterion to pass being the absence of critical errors requiring user intervention: reformat and pick actions for 1–10–100–1,000 tubes filled with 900 μl saline to time the duration of each action; stress tests picking/reformatting over 3,000 tubes per run overnight without close user monitoring; scan performance for input of frozen and non-frozen samples; successful closure of activities during a power interruption (Universal Power Supply test Biostore). No critical errors were observed during any of the tests performed for both the Biostore and the Sample Store. Table 3 shows the time needed to reformat or pick samples by the two automated stores. Generally, reformatting samples takes more time than picking samples (1.2 and 1.3 times quicker for 1,000 samples for Sample Store and Biostore, respectively). As the reformat action moves the tubes from the standard density SBS racks to the high density racks with tightly fitting aperture, the picker module settings have been optimized to maintain accuracy while compromising on speed. Furthermore, the Sample Store (−20°C) handles the tubes about 3 times faster than the Biostore, which again is due to optimized picker module settings for the Biostore to accommodate changes in tray size due to exposure to different temperatures (sample storage at −80°C, sample picking/reformatting at −20°C). No difference in scan performance was found when inputting frozen or non-frozen tubes and overnight runs finalized without any issues.

**Table 3:** Reformat and pick times per store per number of tubes.

	**Sample Store I**		**Biostore I**	
	Reformat time^*^	Pick time^*^	Reformat time^*^	Pick time^*^
1 tube	4	2	5	4
10 tubes	4	2	7	4
100 tubes	7	6	14	9
1,000 tubes	40	33	125	91

* *Time in minutes*.

Additional testing was performed to assess the automated store behavior when exposed to unintended use: generating pick orders for tubes that are not in the store or for duplicated tube barcodes; input Biostore sample trays into the Sample Store and vice versa; input sample racks without barcodes; input sample trays backwards. No criterion for acceptance was set, the resulting observations were used to define the procedure to handle the systems by the users. Apart from the backward tray input, all events were handled to satisfaction by the stores to prevent unintended misuse of the system: orders for tubes not physically present in the store were executed for the tubes where available and remained in “waiting” state until the missing tube was inputted (which successfully finalized the order) or until closure of the order by the user. Duplicated tube requests within one order where ignored and sample racks without barcodes were taken into the store for storage at temperature, but were marked as “problematic” and could only be handled upon intervention by the user. Biostore sample trays could not be accepted into the Sample Store and vice versa. Upon backward input of the sample tray, the tray was lifted and dropped in the input/output module, leading to samples jumping out of the tray and falling into the unit, risking sample loss on the one hand and mechanical obstruction of moving parts on the other. A backward placed tray cannot be detected by the system and is a user-dependent event. It was therefore incorporated as an important part of the internal user training to prevent the event from occurring. Finally, a 2-week period of empty runs mimicking first customers' sample flow was used as formal performance qualification. All acceptance criteria were passed, and no issues were encountered.

**Figure 1:**
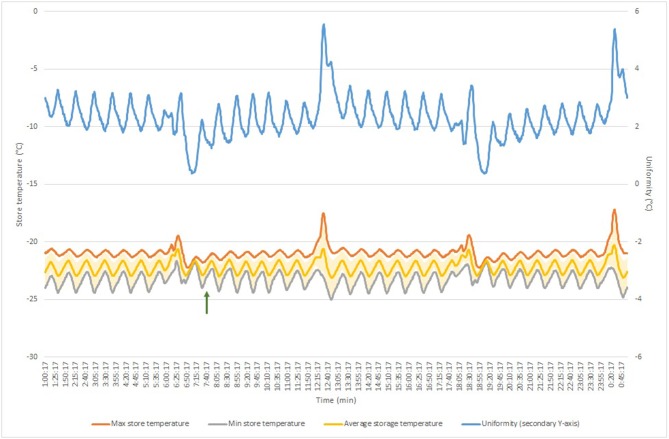
Temperature homogeneity measurement in Sample Store over 24 h measurement period. Green arrows indicate door opening session start. Blue line shows temperature uniformity (displayed on secondary axis); yellow line displays average store temperature (with standard deviation indicated in light-yellow error bars); orange line shows maximum store temperature; gray line shows minimum store temperature.

With the temperature and cooling performance of the automated store being critical to the Biobank requirements, these aspects were also qualified through additional assessments. Temperature homogeneity was assessed based on the Energy Star Program Requirements and the French standard NF X 15-140 using 15 temperature monitoring devices. Results are displayed in Table 4 and Figure 1. The average temperature over the 24 h measurement period was −22.19 and −79.84°C for the Sample Store and the Biostore, respectively. The increased temperature stability of the Sample Store vs. the Biostore (4.67 vs. 1.13°C) is most likely due to the defrost cycling of the Sample Store I occurring every 12 h. Because the Biostore is purged with ultra-dry pressurized air, no frost build-up occurs, omitting the need for defrost cycles which results in a higher temperature stability at a specific location. However, the temperature uniformity within the store at any given time was 2.32°C for the Sample Store I and 8.07°C for the Biostore, indicating a better temperature homogeneity in the Sample Store compared to the Biostore. Warm spots and cold spots were also determined, the locations of which are in line with the layout of the store and the type of cooling and distribution of cold air. Door opening and closing sessions did not measurably impact the stores' temperature stability and uniformity, as evidenced by similar or even lower stability and uniformity values during and after the exercise (Table 4).

**Table 4:** Temperature homogeneity for sample store and biostore.

	**Sample Store**	**Biostore**
Average temperature (24 h)	−22.19 ± 0.77	−79.84 ± 1.99
Max temperature (24 h)	−17.19	−74.90
Min temperature (24 h)	−25.03	−84.10
Peak variance (24 h)	7.84	9.20
Stability (24 h)	4.67	1.13
Uniformity (24 h)	2.32	8.07
Stability (DOC1)	1.69	0.87
Uniformity (DOC1)	2.29	8.07
Stability (DOC2)	1.78	0.28
Uniformity (DOC2)	2.02	8.06
Warm spot	Row 3, column 1, front	Row 5, column 1, front
Cold spot	Row 38, column 30, mid	Row 5, column 13, mid

*DOC, door opening an closing, calculated for a 3 h period starting at the first door opening exercise (DOC1) and calculated for a 3 h period starting 3 h after the last door closing event (DOC2)*.

In addition to the temperature homogeneity, alarm connections to the building monitoring system were successfully tested for all the critical and warning alarms generated by the two automated stores. Furthermore, sustenance of cooling was also determined while the cooling units were running on city water to simulate the backup procedure in case of failure of the chilled water system and during switch to emergency power to simulate power interruptions. In both cases, cooling was sustained as evidenced by normal cycling behavior of the cooling units and stability of the systems' temperature (data not shown). The system qualification and validation phase took about 1 year for 0.5 full-time equivalent of an internal dedicated super-user to complete. These data show that both the cooling and automation aspect of the automated stores meet the requirements of the UZ KU Leuven Biobank qualifying the Sample Store and the Biostore for routine use.

### Implementation

The first actual samples were inputted into the automated stores in February 2018. Performance of the systems is continuously monitored through the evolution of the pick retry and place retry ratios. Typically, these should be below 10% for the Biostore and below 5% for the Sample Store and these thresholds have not been exceeded since first actual input (data not shown). Additionally, the SQAT is used to assess and qualify overall functionality and is performed pre and post preventive maintenance, after unplanned intervention by the user or the technician or as evaluation of the store functionality in case of doubt. Apart from a dysfunctional tray consistently causing reformatting issues, no systematic issues were detected. Errors that could not be addressed by the Biobanks' superuser were swiftly addressed by either remote intervention or on-site visit. Overall, the manufacturer has shown increased attention to the needs of customers within a regulatory environment compared to the earlier stages of the installation. However, the Biobank still needs to keep a close eye to maintain quality of service.

**Figure 2:**
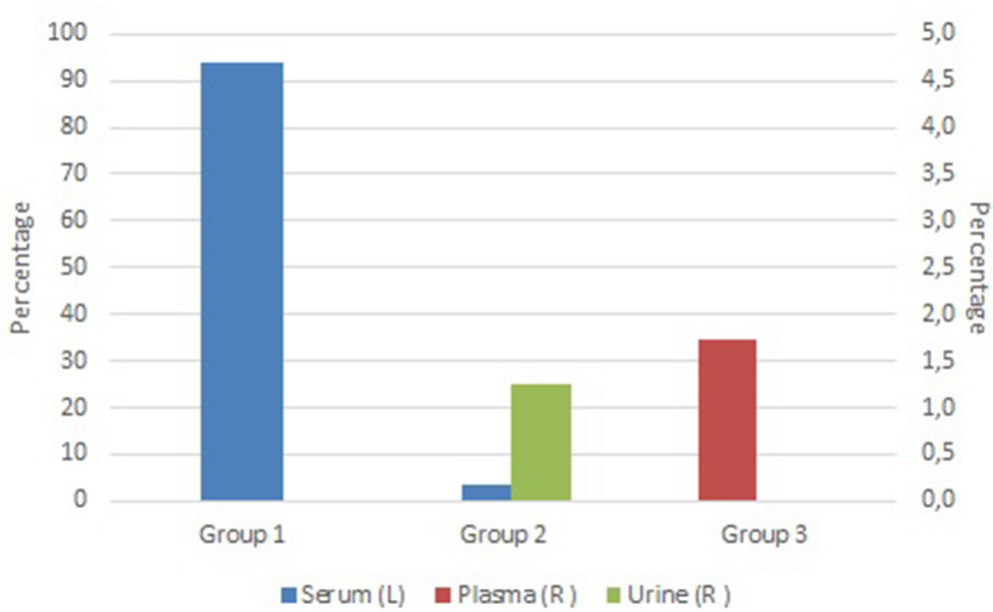
Overview of current sample type distribution stored in the Biostore, clustered by provider. Blue bars show the percentage of serum samples (Y-axis), red bars show the percentage of plasma samples (right Y-axis) and green bars show the percentage of urine samples (right Y-axis).

At the time of writing, the stores hold about 63,000 samples provided by three different research groups. The grand majority of samples are serum samples (97%), followed by plasma (1.7%), and urine samples (1.3%) as shown in Figure 2. This can be explained by the biobanking approach of the different groups. Group 1 has already systematically been collecting and processing various biospecimens from a large patient group at different moments during their treatment since before the year 2000. Group 1 switched to the automated store workflow immediately after final qualification of the equipment. Group 2 and 3 only started to collect and process samples when the automated stores were already in production and target a smaller patient group (Group 2) or focus on small scale projects (Group 3). Transition to automation has decreased actual hands-on sample picking and retrieval time at the expense of the Biobank, but has increased the cost for the research group due to the use of automation-friendly, more expensive tubes. Overall, the transfer from a manual to the automated retrieval process was positively received by the end-users. Some difficulty was encountered for Group 2 and 3 users because of the transfer to tubes only identified by 2D barcodes. However, modification of the research groups' workflow to incorporate scan-based confirmation steps overcame this issue. Step-wise expansion of the number of research groups using the automated stores is ongoing, with another three groups currently in transition. Integration of the automated storage of the Biobank in the clinical laboratories' workflow is planned to start spring 2020. The current utilization rate of the samples is 3.2% which is expected to increase over time when sample follow-up (and significance) increases.

## Discussion

The low reproducibility of biomarker research is a major holdback for the translation of research results to the bedside. Sample integrity has been identified as a key factor that contributes to improved reproducibility. One way of preserving the sample integrity is through automation of processes, thereby significantly reducing the introduction of variation. When handling large numbers of biospecimens in the biobank setting, automation of sample handling and storage is often the method of choice to save labor and improve turnaround times and quality. In this paper, we described the lessons learned during the implementation of two automated frozen sample storage systems at the UZ KU Leuven Biobank (Table 5).

**Table 5:** Overview of the validation approach for the automated stores of the UZ KU Leuven Biobank.

**Phase (duration @ UZ KU Leuven Biobank)**	**Content**	**Who**
Installation qualification (3 years)	Installation by manufacturer and Site Acceptance Test	Manufacturer in presence of user
Operational qualification (1 year)	Intended use: – Develop System Qualification and Acceptance Test – Time to place/pick 1–1000 samples – Time to freeze 1–100 samples – Scan performance of frozen and non-frozen tubes – Stress test by placing/picking >3,000 tubes – Universal Power Supply test during operation	User
	Labware verification: – Determine maximum fill volume and impact on system	
	Unintended use: – Create pick order for duplicate tube ID – Create pick order for tube absent from store – Insert labware with incorrect orientation	
	Cooling: – Determine Temperature homogeneity – Emergency power test and impact on cooling operations – City water backup test and impact on cooling operations	
	Alarm connection Test	
Performance qualification (1 month)	Simulation of intended routine use of the equipment (2 week repeat of estimated sample submissions and requests)	User
Implementation	Routine operation, daily monitoring of PIR and PLR, use of SQAT before/after PM and planned interventions, SQAT after unplanned intervention	User

*PIR, Pick Retry Ratio; PLR, Place Retry Ratio; PM, Preventive maintenance; SQAT, System Qualification and Assessment Test*.

Regulatory standards require a formal qualification and validation of an automated frozen storage system before taking it into production, as well as frequent reassessment of its performance of both the automation as temperature component (13). However, actual method specifications are not provided, nor available in literature for these kinds of systems. The UZ KU Leuven Biobank devised a SQAT test to assess performance of the automated systems and set up a method for temperature homogeneity testing. Although these are tailored to our systems, the underlying concept is applicable to other automated storage systems as most of them share a similar basic concept regarding sample management and storage at a specific temperature (15–17). Our temperature mapping results similar to those reported elsewhere for standard (ultra-low temperature) freezers, even though the storage volume is about 10 times greater (18). Furthermore, door opening and closing actions did not have significant impact to the temperature of the store, whereas this is an important effect in standard freezers, potentially affecting the sample integrity (18). These findings demonstrate the added value of automation in reducing unwanted variation in biospecimens, of major importance for translational research.

The lengthy installation and approval process indicates that automated storage systems are not off-the-shelf products but require substantial adaptations to accommodate site-specific requirements and facilities. Additionally, it involves significant resources from the customer on top of the initial purchase of the equipment, such as assistance from the technical department. It also has to be appreciated that an intense relation has to be set up and maintained between the manufacturer and the biobank during the life-cycle of the product. Our experience and those of others also underline that these appliances will not run out of the box or deliver for the life of the system without ongoing investment (19). Remarkably, the issues we encountered are not site-specific, nor manufacturer-specific: others within the biobanking community have experienced similar difficulties upon implementation of automated storage systems (Europe Biobank Week 2019 pre-conference Automation workshop and GCM & KB, personal communication). Although several reasons underlie these problems, the common ground are mismatched expectations between both manufacturer and client, which require adequate customer service and technical support to be overcome (20). In our experience, appointing one of the biobank personnel as a dedicated super-user was essential to successfully complete the installation and to keep the automated stores operating at optimal performance in collaboration with the manufacturer. Additionally, the implementation of a more structured customer-oriented approach by the manufacturer also contributed significantly to resolving ongoing issues at the UZ KU Leuven Biobank. A similar approach has been reported for large-scale automated compound management systems which emphasized the internal support structure as major factor to maximize return on investment and increase the systems' life-cycle (19).

In conclusion, the centralized UZ KU Leuven Biobank succeeded in the implementation of two automated frozen sample storage systems, which currently hold over 63,000 samples. The majority of ongoing issues have been satisfactorily resolved by the manufacturer in a constructive collaboration with the Biobank. Moreover, additional investments have been made to expand the Sample Store I with an additional module to incorporate additional tube types into the system. As a result, the UZ KU Leuven Biobank is now also able to efficiently support large-scale sample storage for translational research.

## Data Availability Statement

All datasets generated for this study are included in the article/Supplementary Material.

## Author Contributions

LL, KV, and NE devised the conceptual idea. LL executed the work and wrote the manuscript. KV and NE critically reviewed the manuscript.

### Conflict of Interest

The authors declare that the research was conducted in the absence of any commercial or financial relationships that could be construed as a potential conflict of interest.
