# Supplementary material for: Automated Sample Storage in Biobanking to Enhance Translational Research: The Bumpy Road to Implementation
Source: Front Med (Lausanne). 2020 Jan 9;6:309. doi: 10.3389/fmed.2019.00309 (PMC6962113; doi:10.3389/fmed.2019.00309)
Supplement: Supplementary file 1 [file Table_1.DOCX]

**Supplementary Data: Automated sample store structure**

The Sample Store I (-20°C) and the Biostore (-80°C) (Brooks Life Sciences, Manchester, UK) have a similar main design: a main body housing the shuttle and tray conveyor robot for tray selection and one bank at a variable-pitch shelving to store the sample trays (in the Biostore, storage is separated from the robot isle by an insulated tile wall to keep the samples at -80°C while the robot resides at -20°C); an input/output module for tray input/retrieval by user and sample barcode scanning; a cherry picker module at -20°C with a dedicated robot for sample reformat from standard SBS tube racks of 12 x 8 samples per rack (6 racks per tray) to high density trays of 14 x 68 samples per tray and picking (vice versa). Samples are inputted in/outputted from the input/output unit while sitting in standard SBS tubes racks which are put onto dedicated trays. Samples have to go through a reformat routine to be stored at the highest density, picking can only occur from high density trays. Imaging and tube handling are optimized for FluidX 1 ml tubes, with 2D Data Matrix barcoded bottom. Store dimensions and maximum sample capacity are 6m x 1,75m x 2,6m and 700 000 samples for the Sample Store I and 5,5m x 1,9m x 2,7m and 600 000 samples for the Biostore. Mechanical refrigeration is achieved through two redundant, water chilled cooling units per system (one-stage cooling for Sample Store I to obtain -20°C, two-stage cooling for the Biostore to obtain -80°C). Frost, interrupt smooth motion of mechanical parts in a frozen environment, is avoided through 6-hourly defrost cycles in the Sample Store I and by a continuous flow of medical compressed air, subjected to a second drying step resulting in a dew point of below -90°C before being purged into the Biostore. Independent temperature (Sample Store I and Biostore sample storage and robot isle) and humidity sensors (Biostore) as well as alarm connections for the cooling units were installed and connected to the building monitoring system of the University Hospitals Leuven.
